# “Zika is everywhere”: A qualitative exploration of knowledge, attitudes and practices towards Zika virus among women of reproductive age in Iquitos, Peru

**DOI:** 10.1371/journal.pntd.0006708

**Published:** 2018-08-30

**Authors:** Caroline T. Weldon, Amy R. Riley-Powell, Ines M. Aguerre, Rosa A. Celis Nacimento, Amy C. Morrison, Richard A. Oberhelman, Valerie A. Paz-Soldan

**Affiliations:** 1 Tropical Medicine Department, Tulane School of Public Health and Tropical Medicine, New Orleans, Louisiana, United States of America; 2 Global Community Health and Behavioral Sciences, Tulane School of Public Health and Tropical Medicine, New Orleans, Louisiana, United States of America; 3 Asociación Benéfica PRISMA, Lima, Peru; 4 Department of Entomology and Nematology, University of California, Davis, Davis, California, United States of America; National Institutes of Health, UNITED STATES

## Abstract

Zika virus was reported in the rainforest city of Iquitos, Peru in 2016. The potential associations between Zika and fetal neurological disorders were reported extensively in the media regarding neighboring Brazil, and led to great concern about the impact Zika could have on people’s health in Iquitos when it arrived. The aim of this study was to explore the knowledge, attitudes, and preventative practices related to Zika virus and its transmission among women of childbearing age in Iquitos, Peru. Six focus group discussions with 46 women of ages 20–35 from an Iquitos district with confirmed Zika cases were conducted to explore: 1) knowledge of Zika transmission, its symptoms, and treatment, 2) attitudes regarding Zika, including perceptions of risk for and severity of Zika, and 3) preventative practices, including awareness of health promotion activities. Participants were knowledgeable about Zika symptoms and knew it was transmitted by mosquitoes, and about half had heard about the association between Zika and microcephaly, but most lacked knowledge about the associated neurological disorders in adults, its sexual transmission, and ways to prevent infection. They expressed concern for pregnant women exposed to the virus and the impact on the fetus. Participants felt at risk of contracting the Zika virus, yet had not changed preventive practices, possibly in part because their perception of the severity of this disease was low. This study reveals knowledge gaps that could be addressed via health promotion messages that might improve prevention practices to help community members protect themselves from Zika virus during this outbreak.

## Author summary

Zika virus is an arthropod-borne viral disease that has recently caused epidemics in many Latin American countries and is associated with adverse neurologic outcomes and fetal complications. Although the infection is typically mosquito-transmitted, infection is also possible via sexual transmission. This study uses focus group discussions to explore the knowledge, attitudes, and preventive practices of women of childbearing age at risk for Zika in Iquitos, Peru, where Zika had arrived one year earlier (in 2016). We found that most women knew Zika is transmitted via mosquitoes, but few knew it could also be transmitted through sex. We also found that women were aware about the link between Zika and newborn microcephaly, but none mentioned the possible neurologic impact on adults. We also found that very few women had changed their behaviors to prevent getting infected with Zika. By identifying gaps in Zika knowledge, attitudes, and prevention methods, we can improve health promotion and ensure the preventive messages are relevant for those most at risk.

## Introduction

Zika is a vector-borne disease transmitted by a daytime biting mosquito of the *Aedes* species, predominantly *Aedes aegypti* [1]. Common Zika symptoms include rash, mild fever, headache and joint pain [1]. Zika has also been linked to severe neurological disorders, including microcephaly affecting fetuses during pregnancy, contracted through trans-placental transmission, as well as Guillain-Barre syndrome in adults [1]. Since 2015, outbreaks of Zika have been reported in over 60 countries and territories, with more than 750,000 suspected and confirmed cases globally [2].

Zika infections have been reported in eleven of the twenty-five Peruvian departments [3]—both along the coast and in the Amazon basin—and in June of 2016, the first cases of Zika infection were reported in Iquitos, the capital of the Loreto region in the northern Amazon basin [4]. There have been numerous health campaigns in Iquitos to control the *Aedes* vector and improve knowledge and preventive practices related to dengue [5], as all four serotypes have been circulating in Iquitos since 1990 [6] and now, Zika. The health campaigns include emergency fumigation, ongoing larvicide application, and health promotion focused on eliminating unused water containers that may become mosquito oviposition sites [7]. The local regional health authority has led public health campaigns to prevent Zika transmission (via radio, pamphlet, and billboards) [5], but these have not been prolific or remarkably different to existing dengue campaigns, and have not focused on the possible sexual transmission. Condom use is the recommended and most effective method for preventing Zika infection through sexual contact [8], particularly with recent evidence of the important role of sexual transmission of Zika and viral persistence in bodily fluids [9, 10].

Studies that reported low knowledge of what to do to prevent Zika, also showed poor practice to do so [11, 12]. A survey in Brazil conducted with women aged 15–49 during the time of the Zika epidemic found that women with a higher education were more likely to avoid pregnancy due to their knowledge of the association between Zika and microcephaly; higher educated women were also more like to follow preventative measures for Zika infection, such as using protected clothing, applying insecticides, using window screens, and removing standing water breeding sites [13]. A knowledge, attitudes, and practices (KAP) survey related to dengue conducted in Iquitos also found that higher education was associated to more knowledge about dengue and preventive practices that did not involve expenditures, but that higher socio-economic status was associated with preventive practices that required money expenditures [5]. Other recent studies on Zika have suggested the need to strengthen campaigns, communication, and health promotion in communities at risk for Zika [11, 12, 14].

The objective of this study was to examine qualitatively the knowledge, attitudes, and preventive practices associated with Zika and its transmission routes, amongst women of reproductive age, including those who already may be pregnant, in Iquitos, Peru. We focused on examining what *women of reproductive age* know, given the specific risks associated with Zika during pregnancy.

## Materials and methods

### Study setting

Iquitos is the most populated city of the Peruvian Amazon basin (population of 545, 000) [15] and is surrounded by the Amazon, Nanay, and Itaya Rivers, making it only accessible by air or water [16]. The main forms of employment in Iquitos are small businesses in the extractive industries (lumber, fishing, oil), farming, and tourism [17]. This study took place in the Punchana district—one of the four districts in Iquitos. It was selected because of its history of high *Ae*. *aegypti* population densities [6, 16], because Punchana has been previously identified as the district in Iquitos where dengue outbreaks start and having the highest dengue seroprevalence rates overall [6], and because of confirmed presence of Zika virus in this area [3, 4] There is no publication on this yet, but our dengue research team noted that the spatial and temporal progress of Zika transmission after its detection was very rapid in Iquitos (AC Morrison, personal communication, June 2018), and was similar to patterns observed after the introduction of a novel dengue serotype into the city [6, 16]. Since the emergence of Zika in Iquitos, surveillance programs have been put into place to combat the risks associated with infection. There are no cases to date of neurological disease in Peru as a direct cause of Zika, however surveillance remains critical [4, 18].

### Study design

This was a qualitative study comprised of six focus group discussions (FGDs) conducted in June 2017. A focus group discussion guide was developed and applied by our research team (see Appendix A), all proficient or native Spanish speakers, and included a local coordinator from Iquitos to facilitate and ensure cultural understanding. Two of the co-authors (VPS and ACM) have been conducting dengue research in Iquitos for ~35 years combined, including a dengue knowledge, attitudes, and practices survey and FGDs exploring various aspects of dengue transmission and control in the context of Iquitos [5, 19, 20, 21]. The FGD guide was developed by local experts in our research team and focused on qualitatively exploring themes that are common in knowledge, attitudes, and practices (KAP) surveys regarding Zika virus [22, 23]. The main topics explored in the FGDs were: 1) knowledge of Zika transmission, symptoms, and treatment, 2) attitudes regarding Zika, including perceived risk for and severity of Zika, and 3) common preventative practices (e.g., use of repellents or condoms), and health messages received in the community regarding Zika. Because dengue and Zika viruses share a common vector and have similar symptoms, participants were also probed to compare characteristics of Zika and dengue fever symptoms and *Aedes* vector control campaigns in general. The Health Belief Model (HBM) was used to inform the focus group discussion guide, specifically the themes surrounding attitudes towards Zika, by focusing on perceived risk and severity; perceived risk represents a person’s perceived susceptibility of experiencing Zika virus, while perceived severity refers to the beliefs a person holds concerning the seriousness of Zika virus [24].

### Recruitment

Because of our interest in focusing on women’s knowledge and preventive practices, particularly during childbearing years, and because women are more likely to discuss certain topics, such as family planning, in a single sex setting, only women were recruited for FGDs. Purposive sampling was used to recruit women from the Punchana district. This region was selected as there are affiliated research projects in this district and there is known dengue and Zika transmission. Women of prime childbearing years (20–35) were approached near their homes by a local field worker who has worked in this district before and invited those who met inclusion criteria of age and gender, and who were available at the time, to participate in the FGDs. Once enough women were recruited for the FGDs, recruitment stopped. The research team covered transportation costs to and from the FGD sites.

### Data management and analysis

After reviewing the consent form and obtaining verbal consent from each participant, FGDs were audio recorded and later transcribed. There were also two note takers present at each FGD. After each session, the research team met to discuss and summarize the FGDs and compile the notes into one document. These notes were used alongside the transcripts throughout analysis.

A codebook was developed prior to data collection based on themes included in the guide. Once the team had reached saturation—the point where the same topics are emerging in each group [25, 26]–on the main themes being explored, the codebook was edited to reflect additional identified topics. Two researchers coded the discussions using Dedoose version 7.6.13, a web application for managing, analyzing, and presenting qualitative and mixed method research data [27]. Blind coding was used on every other transcription, and then compared, to ensure consistency in code application. Any differences were discussed and addressed before continuing with coding.

### Ethics statement

This study was approved by the Institutional Review Board (IRB) of a local non-governmental organization, Asociación Benéfica PRISMA (CE1425.17), and Tulane School of Public Health and Tropical Medicine (#1040307). Since the FGD with each group of women would only be carried out once and there was no reason to document the participants’ names except if we used a written informed consent, the two IRBs approved the use of verbal consent for participants. Prior to starting the FGDs, all participants gave verbal informed consent to participate and to be audio-recorded. All participants were provided with a copy of the consent document, which included contact information for the IRBs and the PI of the project. All members of the research team completed human subjects training.

## Results

The number of participants in each focus group ranged from 7 to 8, totaling 46 women. All participants were women between the ages of 20 and 35 from the Punchana district. Of the 46 women, two respondents declared that they were pregnant during the FGDs. The results are presented here by the key themes that were identified: 1) knowledge of Zika transmission, symptoms and treatment, 2) attitudes regarding Zika (including perceived risk and severity of Zika), and 3) preventative practices and awareness of health promotion messages of Zika virus.

### Knowledge of Zika virus transmission

All participants from all FGDs knew that infected mosquitos transmit Zika virus, but only one or two participants from each focus group knew that the dengue and Zika virus vector, *Aedes aegypti*, bites during the day. In fact, almost all respondents described the mosquito that transmits Zika to “*sleep during the day*” and “*give problems at night*.” Very few knew about the sexual transmission route: a few participants in three of the six FGDs mentioned this transmission mechanism. In one FGD, a woman suggested that Zika was transmissible through “*sexual relations*,” and the other women laughed and shook their heads in disagreement.

### Knowledge of Zika symptoms

Almost all focus group participants correctly and consistently named the most common symptoms, including rash, mild fever, headache, joint and muscle pain, and red eyes. In all of the FGDs, participants distinguished Zika by its distinctive rash or “*welts*” made up of individual bumps grouped tightly together, which spreads all over the body. The rash was noted as the most common way to differentiate Zika from other similarly presenting illnesses in every FGD. These symptoms were frequently compared to those of dengue fever without prompting from the facilitators. One participant described the differences: “*it was similar to dengue*, *but it was different*, *because it covers you in welts*, *it made you itchy and you felt a fever and the children could not sleep*. *That was the difference*.” When asked about duration of symptoms, responses varied, ranging from 3 to 8 days.

### Knowledge of treatment for Zika

When participants were asked to share their understanding of treatment for Zika, women mentioned that there “*was nothing*” that could be done and it was something “*one has to fight with refreshments [keeping hydrated]*.” Despite this, two or three participants in each FGD mentioned “*paracetamol and chlorphenamine*” (anti-histamine) as a common remedy for Zika infection. The refreshments refer to juices from local fruits including malba (a local plant), lemon, and coconut, primarily used for keeping hydrated and reducing fever. Many participants in three of the six focus groups also discussed covering body surfaces with malba leaves to lower body temperature. Healthcare seeking was rarely reported. One participant went to the health post and was diagnosed with Zika, based on clinical signs, without a blood test. Another pregnant participant described regular blood draws to check for Zika as a part of her prenatal care. However, the majority of women felt that symptoms were primarily controllable by using home remedies and paracetamol (available without prescription) as listed above, and hence, seeking healthcare was not common.

### Perceived risk of Zika

About half of the participants in each FGD knew someone who had experienced Zika virus infection; those who knew someone reported knowing this based on symptom recognition, most commonly the presence of the distinguishable rash. In Iquitos, some laboratory testing was done by the *Instituto Nacional de Salud* (National Institute of Health), but diagnosis was also provided at local health centers without a laboratory diagnosis. A women voiced that there had been “*a lot of Zika*” and they [she and fellow Iquitos residents] felt they were at risk of Zika, but “*the truth is that here in this area*, *as it is a jungle area*, *we are already accustomed to it* [Zika]” because *“there will always be that little animal*, *the mosquito*.*”* And that they had learned not to be afraid of Zika, *“If we are afraid we will be self-conscious in the house*: *I cannot go out*, *I cannot go for a walk because Zika is everywhere*.*”* The women explained that pressures of mosquito-borne illnesses, such as “*going on a walk and suddenly feeling with discomfort that we can contract [Zika]*,” was not a lifestyle they wanted to face. The women appeared to acknowledge the risk of Zika infection, but chose not to live in fear of something they could not control. The fatalistic attitude expressed by the participants was also evident in their head nodding and strong agreement with each other, as noted by the notetakers.

Participants expressed more concern for vulnerable populations, such as children and pregnant women, at risk for contracting Zika virus. Women feared more for children than other family members due to their perceived weaker immune systems and higher susceptibility to high fevers and other symptoms of Zika. When prompted to discuss gender differences relating to Zika, two groups suggested that women were more at risk for mosquito bites than men because their “*defenses are a little lower*” and they spend more time at home, while men are generally in air conditioned rooms at work or in more rural areas with a lower presence of the *Aedes* mosquito. Although none of the participants reported knowing anyone with fetal complications from an infected pregnant mother, in five out of six FGDs, participants expressed concern for pregnant women exposed to Zika because of potential effects on fetal development including “*small heads*” and “*missing limbs*.” One pregnant woman said, *“It mainly affects the babies and the pregnant mothers*…. *let’s say*, *if they get Zika*, *it can be risky for your baby*. *That is*, *it can be born with abnormalities*.*”* There was awareness in five of the six groups of risks for developing fetuses, but no one mentioned neurological disorders associated with adults, such as Guillain-Barre syndrome.

### Perceived severity of Zika

When asked about the severity of Zika compared to other febrile illnesses, including malaria, dengue, and chikungunya, all focus groups but one ranked Zika the least severe of all these febrile illnesses. Participants also stated that *“malaria is the worst and can kill you”* and *“if you get malaria*, *it is worse than dengue and Zika*.*”* The severity of Zika seemed to be ranked lowest on the spectrum of mosquito transmitted diseases in the community.

### Zika prevention practices

The most common responses, stated in all six FGDs, regarding ways to prevent transmission of Zika virus were to use mosquito bed nets and to maintain general household cleanliness. Other reported methods used for mosquito prevention included use of mosquito coils and/or repellent, and emptying water containers to reduce breeding sites. One pregnant woman relayed the advice she had received at a talk at her local health post on preventing transmission of Zika, which included “*not to have [sexual] contact without protection*, *to take care of ourselves*, *to sleep with a mosquito net*, *to have a clean house*.” When prompted, nearly all FGD participants reported that they had not changed their individual or household preventative practices in response to the Zika outbreak. A few women in each FGD explained that preventative practices were meant for general mosquito elimination, not to target a specific vector borne disease.

Prevention of sexual transmission of Zika was only discussed if participants brought up the sexual transmission route. Within the three focus groups that named the sexual transmission route, only two women knew about the recommended waiting period of six months before initiating sexual contact with a Zika infected partner [8]. When prompted, most women reported condom use challenges due to their partners’ preferences: “*they protest*, *but in the end they do it*.” Women said that if they did use condoms, it was primarily for family planning purposes, not for prevention of sexually transmitted infections. One of the two pregnant participants reported avoiding unprotected sex during pregnancy, while the other pregnant participant was not taking any additional precautions to prevent viral sexual transmission during pregnancy. Within our FGD participants, the preferred family planning method was the injection, formally known as the Depo-Provera birth control injection. When asked about family planning, women reported that the conversations with their partners were “*not easy*.” Almost all women strongly reported that they did not want more children and that often children were unplanned.

Another Zika prevention method mentioned in every FGD was the use of insecticide, in the form of emergency fumigation campaigns, administered variably from year to year. These campaigns are run by the *Dirección Regional de Salud* (DIRESA, the regional Ministry of Health), who usually fumigate in the early morning hours when the family is typically at home. The main sentiments FGD participants expressed about the household fumigations was the poor efficacy of the insecticide, or as one said, “*they might be spraying water*, *because instead of disappearing*, *the number of mosquitos are growing*,” and the lack of engagement from the healthcare workers, who often didn’t have conversations with the families.

### Health promotion regarding Zika

Participants reported that health education is not routinely provided alongside fumigation campaign activities. As one participant described “*They don’t know how to share information*. *They speak quickly and leave*.” Another participant described her desire, and the missed opportunity, to learn more:

“They are not going to the houses to teach prevention on how to avoid [Zika] or giving us ways to care for it in the house. For example, yesterday the dengue team came to fumigate. I thought they were also going to give me instructions on what Zika is, no? I … didn’t know what this was. I’m not informed about that.”

This desire was echoed by a number of participants who “*would like to know more*” about Zika prevention. Despite this, the consensus among most participants was that they felt reasonably well informed about Zika, and reported that they primarily relied on television, radio, and verbal and written information from health posts to inform themselves on this topic. Participants in all six focus groups had a very positive attitude towards health messaging in general and they described their media preferences for health information enthusiastically: listening to the radio or television while cleaning, and reading flyers at the health posts during appointments. When asked about who in the family tends to be informed on the topic of health, participants reported that *“the women are more conscious because they have more responsibilities”* for the health of their family and desired to know as much information as possible to prevent their household from infirmity.

## Discussion

The FGDs were completed one year after the first confirmed case of Zika was detected in Iquitos, when the epidemic was at its end. While there was considerable concern about the possible microcephaly or neurological impact of Zika among public health officials, there have been no cases of Zika-related microcephaly reported in Peru to date [4, 18]. In contrast, during the last major dengue outbreak (2010–2011), deaths and high hospitalization rates raised much concern among the population of Iquitos and were highly reported in the news and media. Whilst there was some media exposure to Zika, it was relatively scarce, and was not associated with fatalities or the severe birth defects observed in Brazil [28]. We found that the FGD participants were aware of the emergence of Zika within their region, making them feel at risk, but not fearful, of Zika. They may not be fearful because of the general mild symptom presentation of Zika that was observed in Iquitos (e.g., more rash, but less fever, than with dengue), because of a lack of understanding about the possible neurological effects of the virus, or simply because the communities are accustomed to similar arboviruses. A recent survey on Zika in Honduras revealed a similar lack of knowledge regarding association between Zika and potential neurological outcomes, as well as a low risk perception among the community [12].

Generally, the FGD participants presented little knowledge on the sexual transmission route of Zika and the use of condoms to prevent Zika transmission, despite the evidence of viral presence in semen and vaginal secretions and the ability to sexually transmit the virus [9, 10, 29]. That said, there was one participant that stated that she was avoiding unprotected sex during pregnancy for this reason. In contrast, a qualitative study in Brazil found that women were adapting their family planning due to the Zika outbreak, as encouraged by health services [30]. This change in family planning practice may well be due to the first-hand experiences participants in Brazil had with the neurological effects of the Zika virus. Using condoms during sex, however, is easier said than done: respondents acknowledged available condom resources, but commented that their partners (and men in general) did not like using condoms and needed to be persuaded. Similarly, in another qualitative study about Zika and reproductive decision-making in Iquitos found couples associate condoms with risk, and are not used within committed relationships [30]. The woman’s responsibility for preventing sexual transmission of Zika and men’s aversion to using condoms was also mentioned in a qualitative study in Brazil examining the effect of the Zika virus on family planning practices [31]. Moreover, in previous studies regarding sexual health in Iquitos, researchers described liberal social norms for sex and marriage [32], but insufficient sexual education in secondary schools [33]. One programmatic implication from these findings would be to increase the understanding of the importance of condom use and its role in preventing Zika transmission via sex, at least during an outbreak. Moreover, promoting condom education could have other benefits, including reducing transmission of sexually transmitted infections and preventing unwanted pregnancies—but both men and women need to see this value. Condom use would serve various purposes, since most participants commented that they did not want any more pregnancies.

Despite being aware of Zika in their community, a great majority of FGD participants reported no changes in their preventative practices. Furthermore, some of the preventative practices routinely used for mosquito control reported in FGDs were not effective for preventing the *Aedes aegypti* vector as these practices centered around preventing bites from nighttime biting mosquitos; this was consistent with findings from a survey-based study in Iquitos in 2015 that found that only 18.6% of participants knew that the *Aedes aegypti* bite during the day, despite years of dengue health promotion [5]. Despite low levels of knowledge about the vector, FGD participants were very knowledgeable about the symptoms of Zika and were able to distinguish Zika symptoms from those of dengue, mainly based on the type of rash. This was also consistent with dengue study findings that found low knowledge about the dengue vector and its transmission, but higher knowledge of dengue symptoms [5]. Based on suggestions from FGD participants, existing DIRESA teams that implement larviciding and fumigating campaigns could engage with household members and give short educational sessions during their routine house visits carried out at 3 month intervals. Our FGD participants were keen for new health information to prevent sickness in their homes and suggested that larvicide and fumigation teams are well placed to provide health education.

There was also apparent fatalism demonstrated by the participants in our study regarding their susceptibility to getting Zika and this may be one of the reasons that participants appeared not to make Zika-specific prevention efforts. Zika was perceived as inevitable and there was a certain acceptance of living in a region with high prevalence of Zika and other arboviruses with no cure, only treatment for symptoms. Moreover, the relatively ‘mild’ symptoms reported by the participants—and the fact that there were no reported cases of babies with microcephaly or adults with neurological complications in the news—may have influenced their perception that Zika is not as serious as other, similar illnesses. Finally, despite the perceived higher severity for the fetus, this did not appear to translate into any suggestion of increased or changed preventative methods for pregnant women.

Future health education programs within this region could focus on addressing the fatalism expressed regarding the acquisition of Zika. Other studies which have examined feelings of fatalism suggested that future research and interventions should focus on agency and self-efficacy within specific cultural contexts if they are to address how feelings of fatalism affect care-seeking behaviors [34, 35]. Another way of empowering communities is through participation in health campaigns. One example would be through peer education, in which people are not objects or recipients of educational projects, but participants in this process, who are able to identify their problems and solutions [36, 37]; for example, involving volunteer community members in health promotion initiatives and clean up activities. Our findings suggest that women in these communities are receptive to health education and have expressed a desire to acquire more information regarding Zika. Anecdotally, following completion of the groups many women asked if there would be more FGDs in the future, and were enthusiastic to participate, share their knowledge, and learn more. Research in the Iquitos community has found that people are willing to spend time and money—albeit limited—on controlling vectors to prevent diseases: people report sprinkling and/or mopping petroleum or creoline daily on their floors to try to keep mosquitoes away [20]. That said, the main preventive practice for Zika is vector control, and beyond large scale fumigation and larviciding done by the regional health authorities, community members are limited to controlling breeding sites in a rainforest—and for many, this may seem like a futile task—since personal insect repellents and household insecticide sprays are too expensive for most.

This article should be viewed in light of some limitations. The focus groups were completed until saturation was reached, but the sample size was small and only completed with women from one district of Iquitos. The response rate was not recorded; recruitment consisted of our local research assistant walking through a selected neighborhood in the district and recruiting women between ages 20–35 who were available to meet for the focus group at specific times. In order to elicit opinions on sexual and reproductive health from all participants, future studies could hold a small educational session following the FGDs and then discuss participants’ opinions on sexual health practices and preventative methods once they had acquired the new knowledge. This would have the added benefit of completing some community education sessions alongside data collection.

In conclusion, our findings show that whilst individuals were able to identify some aspects of Zika, such as the symptoms and the mosquito transmission route, as well as the risks for fetal development, the knowledge regarding other aspects of Zika were limited, most notably the sexual transmission and the risk for neurological complications among adults. Perceived severity associated to Zika was consistently low within this study population, which may be partly due to the mild or delayed clinical presentation that was observed in Iquitos. Our findings reveal that the women in this study were especially receptive to health information, and keen to increase their knowledge in order to keep their families healthy. There is an opportunity to improve the knowledge within this at risk population regarding potential neurological outcomes, the sexual transmission pathway, and the most effective preventative methods. Further studies are needed to design and evaluate educational innovations to provide individuals in Zika prevalent regions with quality health information and practices to contribute to the prevention of infection during this epidemic.

## Supporting information

S1 File Focus group guide. This is the focus group guide covering themes associated to knowledge, attitudes, and practices, and sample questions used for each theme.
(DOCX)
