# Supplementary material for: “Zika is everywhere”: A qualitative exploration of knowledge, attitudes and practices towards Zika virus among women of reproductive age in Iquitos, Peru
Source: PLoS Negl Trop Dis. 2018 Aug 30;12(8):e0006708. doi: 10.1371/journal.pntd.0006708 (PMC6135521; doi:10.1371/journal.pntd.0006708)
Supplement: S1 File — This is the focus group guide covering themes associated to knowledge, attitudes, and practices, and sample questions used for each theme. (DOCX) [file pntd.0006708.s001.docx]

**S1 File: Focus group guide**

- Location: Punchana district, Iquitos, Peru
- Participants: Women of child bearing age (20-35 years old)
- Objective: To explore knowledge, attitudes and preventive practices associated with Zika

| **Theme** | **Sample Questions** |
| --- | --- |
| Knowledge of Zika | - Have you heard about Zika? What have you heard about it? Do you know anyone who has had Zika? - What are the symptoms? Anything else? - How does one get Zika? Any other ways? - Is there anything you can do if you get Zika? Any treatment? |
| Attitudes regarding Zika, including perceived risk for getting it, and perceived severity of Zika | - Are people in your communities worried about Zika? Is there anything about Zika they are particularly worried about? - What are people saying about Zika? *(Probe if it does not come up at all: Have they heard of any disabilities associated to Zika?)* - Do you feel at risk of Zika? Do you worry about any family members contracting Zika? - What are the differences and similarities between Zika and dengue? Is one more severe than the other? Which one? Please describe. How about compared to malaria or chikungunya? Explain. - (If aware of risk during pregnancy): Do you feel worried about getting pregnant during this time? |
| Preventative practices, including health promotion | - Are you doing anything to prevent getting Zika? Can you describe some of these things? - Are you doing anything DIFFERENT to what you were doing to prevent other illnesses transmitted by mosquitoes, like dengue? Please describe. - Do you know if friends/family are doing anything different? - Is the Zika prevention, if any, different for women your age than for the rest of the family? - Have you had any discussion with friends or your partner about being pregnant during a Zika outbreak in Iquitos? - (In context of discussion about sexual transmission): Have you or anyone you know started using condoms to prevent Zika as a result? Explore sexual and reproductive preventative practices, if any. - Have you received any health education about Zika? - What are the Zika campaigns focused on? - What would you like to know more about regarding Zika? - What would be the best way to get this information to you and people in your community? Is there anyone the messages should focus on? |
